# Synergistic cytotoxicity of fludarabine, clofarabine, busulfan, vorinostat and olaparib in AML cells

**DOI:** 10.3389/fonc.2023.1287444

**Published:** 2023-11-23

**Authors:** Benigno C. Valdez, Bin Yuan, David Murray, Jeremy Leon Ramdial, Yago Nieto, Uday Popat, Xiaowen Tang, Borje S. Andersson

**Affiliations:** ^1^ Department of Stem Cell Transplantation and Cellular Therapy, University of Texas MD Anderson Cancer Center, Houston, TX, United States; ^2^ Department of Oncology, University of Alberta, Edmonton, AB, Canada; ^3^ Department of Hematology, The First Affiliated Hospital of Soochow University, Jiangsu Institute of Hematology, Suzhou, China

**Keywords:** alkylating agent, nucleoside analog, HDAC inhibitor, PARP inhibitior, stem cell transplant (SCT), pre-transplant regimen

## Abstract

Combinations of nucleoside analog(s) and DNA alkylating agent(s) are used for cancer treatment as components of pre-transplant regimens used in hematopoietic stem cell transplantation. Their efficacies are enhanced by combining drugs with different mechanisms of action, which also allows a reduction in the individual drug dosages and thus potentially in toxicity to the patient. We hypothesized that addition of SAHA and olaparib, an HDAC- and a PARP-inhibitor, respectively, to the established combination of fludarabine, clofarabine and busulfan would enhance AML cell cytotoxicity. Exposure of the AML cell lines KBM3/Bu250^6^, MV4-11, MOLM14 and OCI-AML3 to the 5-drug combination resulted in synergistic cytotoxicity with combination indexes < 1. Increased protein acetylation and decreased poly(ADP-ribosyl)ation were observed, as expected. Activation of apoptosis was suggested by cleavage of Caspase 3 and PARP1, DNA fragmentation, increased reactive oxygen species, and decreased mitochondrial membrane potential. The reduction in poly(ADP-ribosyl)ation was independent of caspase activation. Several proteins involved in DNA damage response and repair were downregulated, which may be contributing factors for the observed synergism. The increased phosphorylation of DNAPKcs suggests inhibition of its kinase activity and diminution of its role in DNA repair. A similar synergism was observed in patient-derived cell samples. These findings will be important in designing clinical trials using these drug combinations as pre-transplant conditioning regimens for AML patients.

## 1 Introduction

Preclinical and clinical studies have demonstrated the synergistic cytotoxicity of fludarabine (Flu), clofarabine (Clo), and busulfan (Bu) towards acute myeloid leukemia (AML) cells (1–5). The antineoplastic activities of Bu, a DNA alkylating agent, and the two nucleoside analogs Flu and Clo involve different mechanisms; Bu forms DNA adducts/crosslinks while Flu and Clo become incorporated into newly replicating DNA strands during DNA synthesis, and they transiently arrest the cell cycle at G2- and S-phase, respectively (6, 7). Both processes trigger a chain of events including formation of DNA strand breaks and subsequent activation of pro-apoptotic pathways (7–9). Combinations of DNA alkylator(s) and nucleoside analog(s) are effective pre-transplant regimens for hematopoietic stem cell transplantation (HSCT) for AML patients, provided that the drugs are sequenced properly, with the nucleoside analog(s) being administered before the alkylating agent (1, 7, 10).

The cytotoxicity of [Flu+Clo+Bu] is enhanced with histone deacetylase (HDAC) inhibitors (11–14), hypomethylating agents (13), BCL-2 inhibitors (15), and FLT3 inhibitors (16). HDAC inhibitors (HDACi) such as SAHA/vorinostat restore appropriate gene expression, resulting in induction of cell differentiation, cell cycle arrest and apoptosis (17). Despite their preclinical efficacy, HDACi do not seem to have high clinical effectiveness as monotherapy, and potentially more effective anti-tumor activity is observed when they are combined with other anti-cancer drugs (18–20). In this context, the differential effects of HDACi on the expression of cellular drug transporters must also be considered before applying them in combination chemotherapy, e.g., they are known to decrease MRP1 protein and increase MDR1 protein levels in human hematologic cancer cell lines (21). Such mechanisms may partly explain the lack of clinical efficacy when the HDACi romidepsin was combined with MDR1 ligands such as doxorubicin or vincristine (22, 23).

The efficacy of HDACi in combination chemotherapy may be attributed in part to its ability to induce DNA double-strand breaks (DSBs); in fact, HDACi-mediated changes in chromatin structure directly activate the DNA damage response (24, 25). HDACi affect the acetylation status of proteins involved in different DNA repair pathways and may have an impact on the genomic instability of cancer cells (26).

Another class of drugs which affect genomic instability are the poly(ADP-ribose) polymerase (PARP) inhibitors. PARP enzymes catalyze protein poly(ADP-ribosyl)ation (PARylation); they bind to DNA strand breaks, self-ribosylate, and recruit and PARylate DNA repair proteins (27). Inhibition of PARP enzymes compromises the localization, stability and activity of chromatin factors including histones, topoisomerases and DNA repair proteins, thereby affecting the DNA damage response (28, 29).

HDACi interact significantly with PARP inhibitors (PARPi) (30, 31). We recently showed that HDACi inhibit protein PARylation and exhibit synergistic cytotoxicity with chemotherapeutic agents such as gemcitabine (Gem), Bu and melphalan (Mel) in lymphoma cell lines (31). Clinically, the combination [Gem+Bu+Mel+SAHA+Olaparib (Ola)] provided ~90% event-free and overall survival rates for lymphoma patients undergoing autologous HSCT (32). These results prompted us to expand this rationale into myeloid malignancies and determine the synergistic cytotoxicity of an alkylating agent, nucleoside analog(s), HDACi, and PARPi in AML cells. Using the proven clinical efficacy of [Flu+Clo+Bu] in AML as a basis, this three-drug combination was combined with the HDACi SAHA/vorinostat and the PARPi, Ola. We now report the synergistic cytotoxicity of these five drugs in AML cell lines and cell samples derived from patients with acute leukemia. The results provide another level of mechanistic insight into the previously reported observations on the HDACi- and PARPi-mediated inhibition of DNA repair in hematologic cancers and its potential exploitation for therapeutic purposes in allogeneic stem cell transplantation.

## 2 Methods

### 2.1 Cell culture

The AML cell lines MV4-11, MOLM14 and OCI-AML3 were obtained from Dr. Michael Andreeff at the University of Texas MD Anderson Cancer Center (UTMDACC). The busulfan-resistant KBM3/Bu250^6^ (KBu) cell line was established in our laboratory by serial exposure of KBM3 cells (33) to increasing concentrations of Bu. All cells were cultured in RPMI 1640 (Mediatech, Inc., Manassas, VA) with 10% heat-inactivated fetal bovine serum (FBS: Gemini Bio Products, West Sacramento, CA), 100 U/ml penicillin and 100 μg/ml streptomycin (Mediatech) at 37°C in a fully humidified atmosphere of 5% CO_2_ in air.

### 2.2 Patient samples

**Figure 1:**
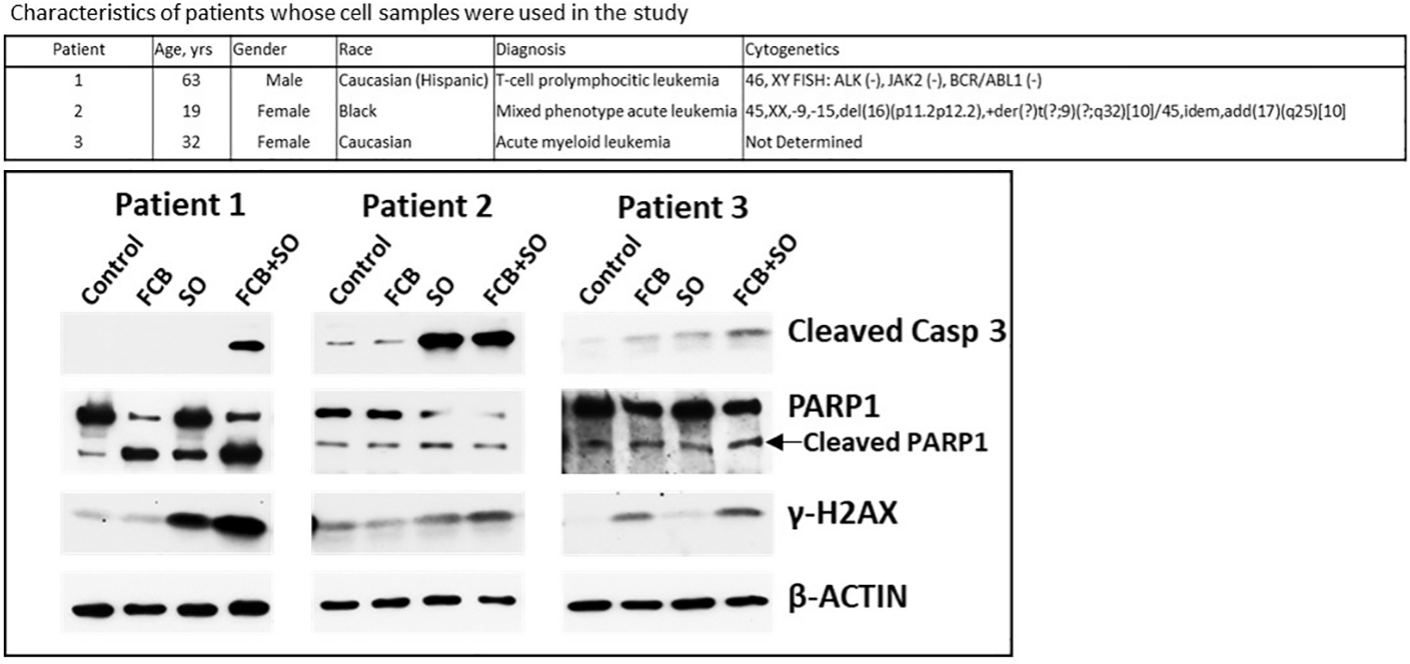
Effects of drugs on molecular markers of apoptosis in patient-derived cell samples. Mononuclear cells were isolated from peripheral blood of patients with leukemia (upper panel) and exposed to the indicated drugs for 48 h prior to analysis by Western blotting (lower panel). F: 0.15 µM fludarabine; C: 15 nM clofarabine; B: 15 µg/ml busulfan; S: 0.6 µM SAHA/vorinostat; O: 7 µM olaparib. Casp, caspase.

Leukemia cell samples were isolated from patients’ peripheral blood using lymphocyte separation medium (Mediatech) and incubated in suspension in the RPMI 1640 medium described above. Patient 1 had T-cell prolymphocytic leukemia (T-PLL), patient 2 had mixed phenotype acute leukemia and patient 3 had AML, as shown in **Figure 1**. Patient samples were collected after obtaining written informed consent, and all studies using these samples were performed under a protocol approved by the Institutional Review Board of the UTMDACC, in accordance with the Declaration of Helsinki.

### 2.3 Drugs

Fludarabine (Flu), clofarabine (Clo), SAHA/vorinostat, olaparib (Ola), NU7741, LTURM34 and AZD7648 were purchased from Selleck Chemicals LLC (Houston, TX), and busulfan (Bu) was obtained from Sigma-Aldrich Chemical Sciences Corporation (St. Louis, MO). Z-VAD-FMK was purchased from Cayman Chemical Co. (Ann Arbor, MI). Drug stock solutions were dissolved in dimethyl sulfoxide (DMSO) and the final concentration of DMSO was <0.08% by volume, a level that does not induce differentiation of these cell lines.

### 2.4 Cell proliferation and cell death assays

Cell proliferation assays were done in triplicate using 96-well plates. Cell aliquots (100 µl of 0.5 X 10^6^ cells/ml) were analyzed for proliferation using the 3-(4,5-dimethylthiazol-2-yl)-2,5-diphenyl tetrazolium bromide (MTT) assay as previously described (34). The inhibition of cell proliferation after a 48-h drug exposure was determined relative to the control cells exposed to solvent alone. Graphical analyses including calculations of IC_10_–IC_20_ values (the concentration of drug required for 10–20% growth inhibition) were done using Prism 5 (GraphPad Software, San Diego, CA). Programmed cell death was determined by flow cytometric measurements of phosphatidylserine externalization with Annexin-V-FLUOS (Roche Diagnostics, Indianapolis, IN) and 7-aminoactinomycin D (BD Biosciences, San Jose, CA) using a Muse Cell Analyzer (MilliporeSigma, St. Louis, MO).

Drug combination effects were estimated based on the combination index (CI) values calculated using the CompuSyn software (Combo Syn, Inc., Paramus, NJ). This program was developed based on the median-effect method: CI < 1 indicates synergy, CI ≈ 1 is additive, and CI > 1 suggests antagonism.

### 2.5 Western blot analysis

Cells were exposed continuously to drug(s) for 48 h, harvested and washed with cold phosphate-buffered saline (PBS). Cells were lysed with lysis buffer (Cell Signaling Technology, Danvers, MA). Total protein concentrations in the cell lysates were determined using a BCA Protein Assay kit (Thermo Fisher Scientific, Rockford, IL). Western blot analysis was done by separating protein extracts on polyacrylamide-SDS gels and blotting onto nitrocellulose membranes (Bio-Rad, Hercules, CA). Immunoblot analyses were done using the Immobilon Western Chemiluminescent HRP Substrate (MilliporeSigma). The sources of the antibodies and their optimum dilutions are available upon request. The β-actin protein was used as an internal control.

### 2.6 Determination of the level of PARylation

The levels of total PARylated proteins were determined by Western blot analysis (as described above) and enzyme-linked immunosorbent assay (ELISA) using the poly(ADP-ribose) ELISA kit from Cell Biolabs, Inc. (San Diego, CA). The monoclonal anti-PAR antibody used for Western blotting was obtained from R&D Systems, Inc. (Minneapolis, MN). The antibody is specific for PAR polymers 2 to 50 units long, but does not recognize structurally related RNA, DNA, ADP-ribose monomers, NAD, or other nucleic acid monomers.

### 2.7 Analysis of reactive oxygen species

Cells were analyzed for production of ROS using CM-H2DCFDA (5-(and-6)-chloromethyl-2′,7′-dichlorodihydrofluorescein diacetate, acetyl ester), an ROS indicator that diffuses into cells where it is oxidized to a fluorescent product (Thermo Fisher Scientific). Briefly, cells were aliquoted (0.4 ml) into tubes and CM-H2DCFDA (3 μl of 0.12 mM solution in DMSO) was added. Cells were incubated at 37°C for 1 h and immediately analyzed with a Gallios Flow Cytometer (Beckman Coulter, Inc., Indianapolis, IN) using excitation/emission wavelengths of 492/520 nm. Arithmetic means of the fluorescence intensities were compared and the relative fold increase in ROS production was calculated.

### 2.8 Analysis of mitochondrial membrane potential

An MMP kit (Cayman Chemical Co.) was used to determine changes in the MMP using the JC-1 (5,5′,6,6′-tetrachloro-1,1′,3,3′-tetraethylbenzimidazolylcarbocyanine iodide) reagent. Cells to be analyzed were aliquoted (0.4 ml) into flow cytometry tubes. Diluted (1:10 with cell growth medium, 3 μl) MMP-sensitive fluorescent dye JC-1 was added to each tube, incubated at 37°C for 20 min, and analyzed by flow cytometry (λex = 488 nm) using the 530 nm (FL-1 channel, green) and 585 nm (FL-2 channel, red) band-pass filters. Healthy cells with functional mitochondria and high MMP exhibit red fluorescence (aggregated JC-1), whereas cells with disrupted mitochondria and low MMP show green fluorescence (monomeric JC-1).

### 2.9 Statistical analysis

Results are presented as the mean ± standard deviation of at least three independent experiments and statistical analysis was performed using Student’s paired t-test with a two-tailed distribution (Microsoft Excel, Redmond, WA, USA). A *P* value of <0.05 was considered statistically significant.

## 3 Results

### 3.1 [Flu+Clo+Bu+SAHA+Ola] combination shows synergistic cytotoxicity in AML cells

**Figure 2:**
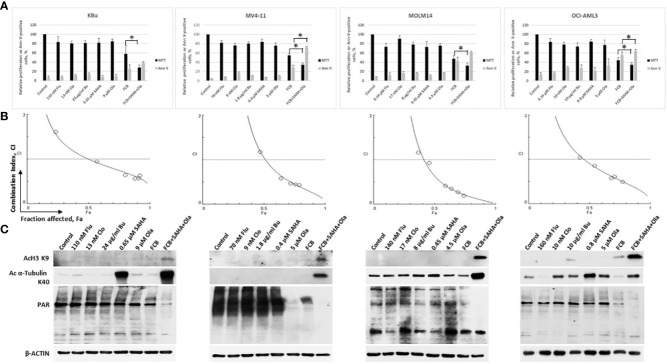
Cytotoxicity of fludarabine (Flu/F), clofarabine (Clo/C), busulfan (Bu/B), vorinostat (SAHA/S), and olaparib (Ola/O) in AML cell lines. Cells were exposed to the indicated concentrations of the drugs, alone or in combination, for 48 h prior to determination of relative cell proliferation by the MTT assay and apoptosis by the Annexin V (Ann V) assay **(A)**. The results are averages of three independent experiments and each experiment was done in duplicate. *P* values less than 0.05 are indicated by an asterisk (*). **(B)** To determine drug synergism, cells were exposed to various drug combinations at constant ratio concentrations for 48 h prior to the MTT assay. The relationships between the calculated combination indexes (CI) and fraction affected (Fa) are shown; CI <1.0 indicates synergism. The graphs are representatives of two independent experiments. **(C)** After 48-h drug exposure, cells were lysed, boiled and analyzed for the levels of protein acetylation and poly(ADP-ribosyl)ation by Western blotting.

Cells were exposed to individual drugs to calculate their IC_10-20_ values, which were used in the drug combination experiments to determine their effects on cell proliferation and apoptosis. Exposure of KBu, MV4-11, MOLM14, and OCI-AML3 cells to Flu, Clo, Bu, SAHA or Ola resulted in 9-28% growth inhibition. [Flu+Clo+Bu] inhibited growth by 42%, 45%, 52%, and 56% in KBu, MV4-11, MOLM14, and OCI-AML3 cells, respectively (**Figure 2A**). Addition of SAHA and Ola to the three-drug combinations inhibited cell proliferation by 66%-71% versus control (**Figure 2A**). The [Flu+Clo+Bu+Ola] combination decreased cell proliferation more than [Flu+Clo+Bu+SAHA] in AML cells (data not shown). All of these results correlate with the corresponding Annexin V data. The [Flu+Clo+Bu] combination caused ~28%-56% cell death as indicated by Annexin V positivity. Exposure to the [Flu+Clo+Bu+SAHA+Ola] combination increased Annexin V positivity to 38%-72% of control (**Figure 2A**). To test for a synergistic interaction, cells were exposed to different concentrations of the individual drugs or to the five-drug combination at a constant concentration ratio, and the MTT assay was performed. The calculated CI at increasing drug effects was graphically analyzed. **Figure 2B** shows combination index (CI) values <1 at fraction affected (Fa) > 0.5 in all cell lines tested, suggesting synergism for drug concentrations where potentially clinically relevant cytotoxicity was apparent. The graphs also suggest that drug antagonism (CI > 1) may exist at lower drug concentrations where lower Fa values were observed, and which resulted in relatively little cytotoxicity (**Figure 2B**).

SAHA is known to inhibit histone deacetylases, and Ola is a potent inhibitor of PARP. We, therefore, sought to determine their effects on the acetylation of histone 3 and α-tubulin as well as on the PARylation of proteins. Exposure of AML cells to low levels of SAHA (0.4 µM – 0.8 µM) did not cause any significant increase in the level of AcH3K9 but resulted in a modest increase in the level of Ac α-tubulin K40 (**Figure 2C**). The [Flu+Clo+Bu] combination had minimal effect on the acetylation of histone 3 and α-tubulin; addition of [SAHA+Ola] to this combination increased the acetylation of both proteins (**Figure 2C**). Olaparib alone at the concentrations used here (4.5 µM – 9 µM) did not decrease the PARylation levels except in MV4-11 cells; its addition to [Flu+Clo+Bu+SAHA] combination, however, markedly decreased the levels of PAR in all four AML cell lines tested (**Figure 2C**). These observations are consistent with the previously reported synergistic interactions between HDAC and PARP inhibitors (27, 30, 31); addition of [Flu+Clo+Bu] apparently enhances the inhibition of PARylation.

### 3.2 [Flu+Clo+Bu+SAHA+Ola] induces apoptosis

**Figure 3:**
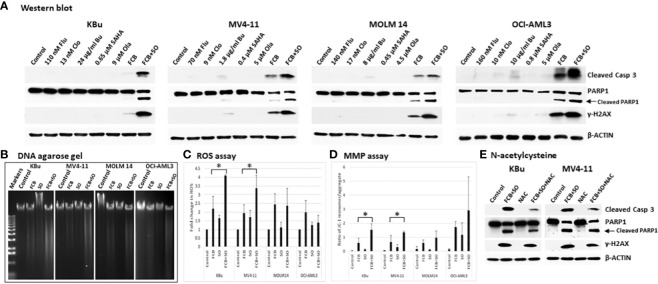
The five-drug combinations strongly activate apoptosis. Cells were exposed to the indicated drugs for 48 h, harvested, and analyzed for **(A)** molecular markers of apoptosis by Western blotting, **(B)** cleavage of DNA by agarose gel electrophoresis, **(C)** reactive oxygen species (ROS) production, and **(D)** changes in mitochondrial membrane potential (MMP). The results in panels **(C, D)** are averages of three independent experiments. *P* values less than 0.05 are indicated by an asterisk (*). **(E)** Cells were pre-exposed to 5 mM N-acetylcysteine (NAC) for 1 h prior to addition of the 5-drug combination and cells were analyzed by Western blotting after 48 h. Casp: Caspase; other abbreviations are the same as in **Figure 2**.

Whether the observed increases in Annexin V positivity (**Figure 2A**) might be associated with apoptotic cell death was assessed by analyzing the cleavage of Caspase 3 and PARP1, which are indicators of apoptosis. While [Flu+Clo+Bu] caused cleavage of Caspase 3 and PARP1, addition of SAHA and Ola to this triple-drug combination markedly enhanced their cleavage (**Figure 3A**). Phosphorylation of histone 2AX also increased in cells exposed to [Flu+Clo+Bu] and even more so after [Flu+Clo+Bu+SAHA+Ola], suggesting DSB formation and/or activation of the DNA-damage response, consistent with the observed cleavage of genomic DNA as shown by agarose gel analysis (**Figure 3B**), which was probably due to caspase-mediated activation of nuclear DNases (35).

### 3.3 [Flu+Clo+Bu+SAHA+Ola] induces production of ROS

To further identify possible mechanisms of apoptosis activation, we examined the production of ROS in AML cells exposed to [Flu+Clo+Bu+SAHA+Ola]. Flow cytometric analysis showed an ~2-fold increase in ROS levels in KBu and MV4-11 cells exposed to [Flu+Clo+Bu] versus control cells, which further increased to ~3.5- to 4-fold versus control cells after exposure to the [Flu+Clo+Bu+SAHA+Ola] 5-drug combination (*P* < 0.05 in both cell lines); in contrast, no significant change (*P* > 0.05 in both cell lines) was observed when SAHA and Ola were added to the [Flu+Clo+Bu] combination in MOLM14 and OCI-AML3 cells (**Figure 3C**). Mitochondrial membrane potential (MMP) decreased in all four cell lines after exposure to [Flu+Clo+Bu+SAHA+Ola] whereas more modest effects were seen with the [Flu+Clo+Bu] combination, as suggested by the relative increases in monomer JC-1/aggregate JC-1 ratio (**Figure 3D**); the increase in the JC-1 ratio between [Flu+Clo+Bu] and [Flu+Clo+Bu+SAHA+Ola] treatments was statistically significant (*P* < 0.05) in KBu and MV4-11 cell lines. The decrease in MMP may cause pro-apoptotic factors to leak from the mitochondria and activate caspases. Indeed, the level of active Caspase 3 (based on its cleavage) also increased more in cells exposed to [Flu+Clo+Bu+SAHA+Ola] than to [Flu+Clo+Bu] (**Figure 3A**). This Caspase 3 activation and decreased MMP closely paralleled the triggering of apoptosis as shown by the Annexin V assay (**Figure 2A**). Overall, cell exposure to [Flu+Clo+Bu+SAHA+Ola] induced ROS production and decreased MMP, potentially contributing to the leakage of pro-apoptotic factors and activation of caspases, and committing cells to apoptosis as shown by the cleavage of Caspase 3 and PARP1, as well as by genomic DNA fragmentation (**Figures 3A, B**). The conclusion that ROS production and mitochondrial dysfunction are in part responsible for apoptosis induction was supported by the observation that treating KBu or MV4-11 cells with 5 mM N-acetylcysteine, an ROS scavenger, for 1 h prior to the 5-drug combination treatment did not completely inhibit apoptosis as shown by partial decrease in the levels of cleaved caspase 3 and γ-H2AX (**Figure 3E**).

### 3.4 The inhibition of PARylation mediated by [Flu+Clo+Bu+SAHA+Ola] does not depend on caspase activation

**Figure 4:**
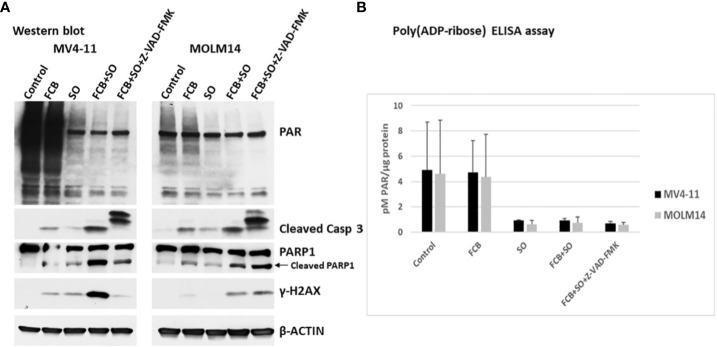
The inhibition of poly(ADP-ribosyl)ation by [Flu+Clo+Bu+SAHA+Ola] is caspase independent. Cells were exposed to the indicated drug combinations with or without 40 µM caspase inhibitor Z-VAD-FMK (added 30 min before the other drugs) for 48 h prior to **(A)** Western blot analysis and **(B)** Poly(ADP-ribose) ELISA assay. The results in panel B are averages of two independent experiments. Abbreviations and drugs concentrations are the same as in **Figure 2**. Casp, Caspase.

Since Caspase 3 was activated by the [Flu+Clo+Bu+SAHA+Ola] combination, we wished to determine if the observed inhibition of PARylation was caspase-dependent. To this effect, cells were exposed to an irreversible pan-caspase inhibitor, Z-VAD-FMK, 30 min prior to addition of the five-drug combination and analyzed by Western blotting and ELISA. **Figure 4A** shows PARylation of multiple proteins in both the control and [Flu+Clo+Bu] treatment groups. The [SAHA+Ola] and [Flu+Clo+Bu+SAHA+Ola] combinations significantly inhibited PARylation; however, addition of Z-VAD-FMK did not affect this drug-mediated inhibition (**Figure 4A**). Again, significant cleavage of Caspase 3 and PARP1 was observed in cells exposed to [Flu+Clo+Bu+SAHA+Ola] and addition of Z-VAD-FMK inhibited Caspase 3 cleavage in MV4-11 and MOLM14 cells but had little effect on PARP1 cleavage (**Figure 4A**). Interestingly, the phosphorylation of histone 2AX seen after [Flu+Clo+Bu+SAHA+Ola] treatment was markedly inhibited by Z-VAD-FMK in MV4-11 cells but not in MOLM14 cells (**Figure 4A**). Quantitative determination of the levels of PARylation by ELISA provided similar results (**Figure 4B**), suggesting that the observed inhibition of PARylation is caspase independent.

### 3.5 [Flu+Clo+Bu+SAHA+Ola] has similar synergistic effects in patient-derived leukemia cells

To assess the potential clinical extension of our results, cells from patients with leukemia were exposed to [Flu+Clo+Bu], [SAHA+Ola], or [Flu+Clo+Bu+SAHA+Ola] and analyzed by Western blotting. Increased cleavage of Caspase 3 was apparent in patient samples 1 and 3 exposed to the five-drug combination (patient 2 had protein loading problems as indicated by unequal levels of β-ACTIN), suggesting robust activation of the apoptosis pathway; a modest cleavage of PARP1 was observed in cells exposed to the 5-drug combination (**Figure 1**). Phosphorylation of histone 2AX was also observed in cells exposed to the 5-drug combination, suggesting DSB formation and/or activation of the DNA-damage response. These findings suggest a synergism of [Flu+Clo+Bu+SAHA+Ola] in cells derived from patients with leukemia involving mechanisms similar to those seen in the cultured cell lines. Additional patient samples may need to be analyzed to confirm the clinical relevance of these results.

### 3.6 [Flu+Clo+Bu+SAHA+Ola] combination affects the levels and phosphorylation of proteins involved in DNA damage response and repair

**Figure 5:**
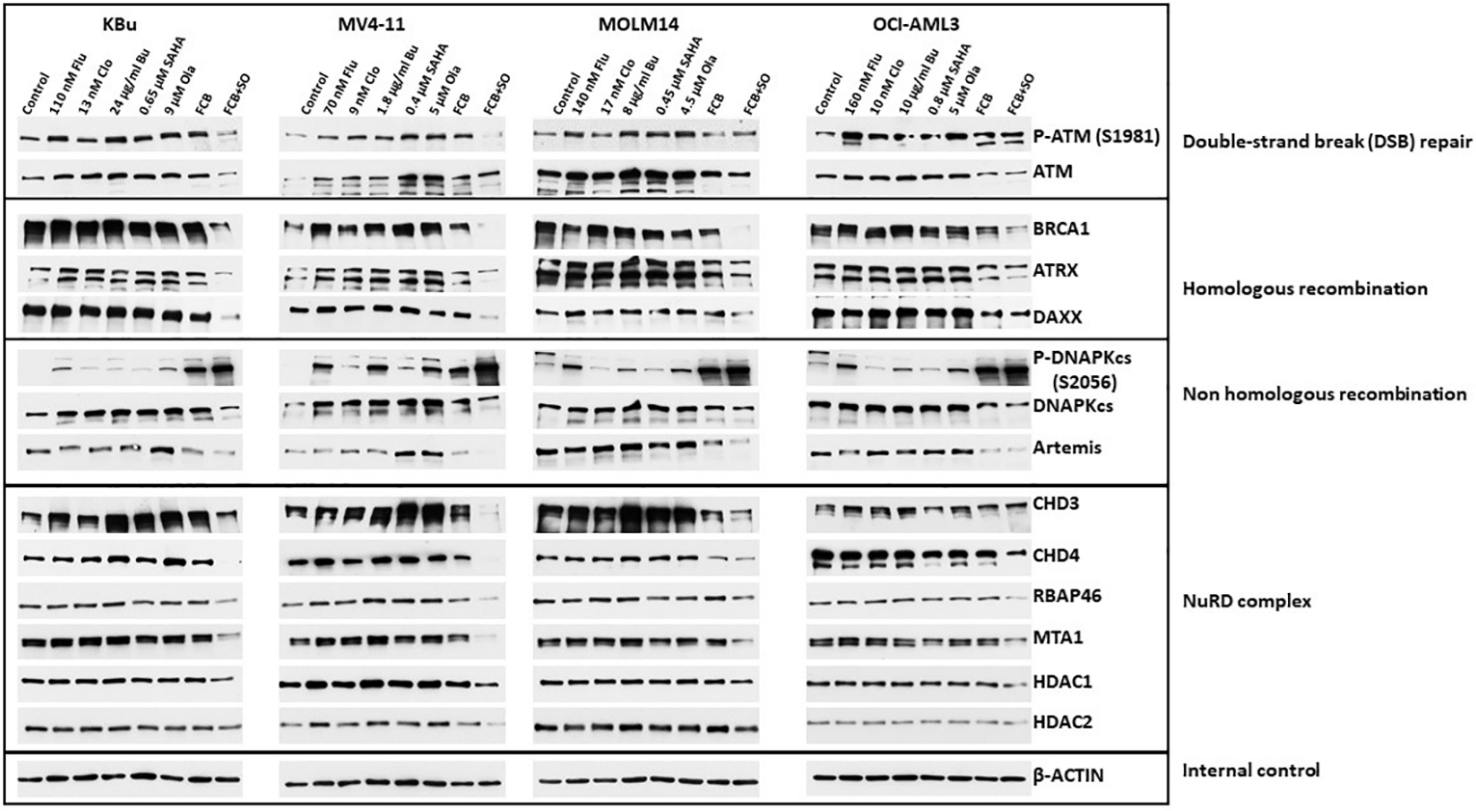
Effects of drugs on the levels and phosphorylation status of various proteins involved in DNA repair/DNA damage response. Cells were exposed to the indicated drug concentrations for 48 h prior to analysis by Western blotting. NuRD, Nucleosome Remodeling and Deacetylase.

Acetylation and PARylation are known to occur in some proteins involved in DNA repair (27, 36). These post-translational modifications affect the stability of the proteins, as previously shown for UHFR1 and BRCA1 (37, 38). We, therefore, examined the effects of Flu, Clo, Bu, SAHA and Ola, individually or in combinations, on DNA damage response protein levels (total and phosphorylated) in AML cell lines. In general, the levels of phosphorylated and pan ATM (which functions in DNA DSB repair and cell cycle checkpoint activation) decreased in cells exposed to the five-drug combination; the level of BRCA1 (which functions in homologous recombination (HR) repair) greatly decreased (**Figure 5**). ATRX is a chromatin remodeling protein involved in HR (39) while DAXX has multiple functions in human cells, including regulation of DNA repair (40). The levels of both proteins decreased in AML cells exposed to the five-drug combination (**Figure 5**). While the level of the non-homologous end joining (NHEJ) repair protein DNAPKcs decreased in cells exposed to [Flu+Clo+Bu+SAHA+Ola], its phosphorylation at serine 2056 dramatically increased; the level of another NHEJ protein, Artemis, also decreased (**Figure 5**).

The NuRD complex is involved in chromatin remodeling and deacetylation processes (41) and plays a key role in the cellular DNA damage response by regulating DNA damage signaling and repair events (42). The levels of the CHD3, CHD4, RBAP46, and MTA1 subunits of NuRD decreased in all cell lines exposed to [Flu+Clo+Bu+SAHA+Ola]; the HDAC1 subunit also modestly decreased, but HDAC2 was only minimally affected (**Figure 5**).

**Figure 6:**
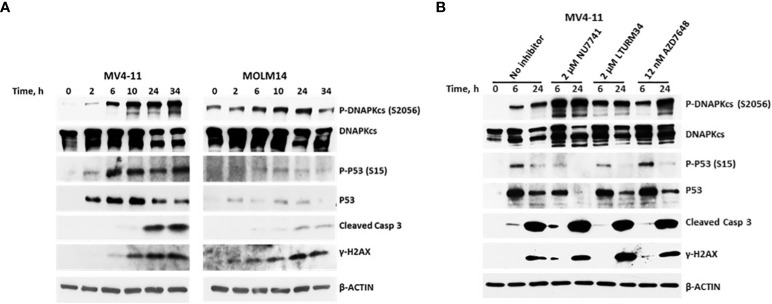
Kinetics of the phosphorylation of the DNA-dependent protein kinase catalytic subunit (DNAPKcs). **(A)** Cells were exposed to five-drug combinations for various times and analyzed by Western blotting. MV4-11 cells: 70 nM fludarabine, 9 nM clofarabine, 1.8 µg/ml busulfan, 0.4 µM SAHA and 5 µM olaparib. MOLM14 cells: 140 nM fludarabine, 17 nM clofarabine, 8 µg/ml busulfan, 0.45 µM SAHA and 4.5 µM olaparib. **(B)** MV4-11 cells were pre-exposed to the indicated DNAPK inhibitors for 1 h prior to addition of [Flu+Clo+Bu+SAHA+Ola] and analyzed by Western blotting after 6 h and 24 h. Casp, caspase.

Of all the DNA damage response/repair protein events analyzed, the increased phosphorylation of DNAPKcs at serine 2056 was most intriguing. We, therefore, analyzed the kinetics of its phosphorylation. **Figure 6A** shows a marked increase in the level of P-DNAPKcs (S2056) within 6 h of exposure to [Flu+Clo+Bu+SAHA+Ola], similar to the kinetics of P53 phosphorylation at serine 15 and phosphorylation of histone 2AX in the MV4-11 and MOLM14 cell lines. These results are consistent with the reported function of DNAPKcs, which is to control the repair of the broken DNA ends and transmit the damage signal through P53 protein to induce cell cycle arrest and apoptosis (43). Whereas the level of P-DNAPKcs increased with drug treatment, the level of unphosphorylated DNAPKcs decreased within 24 h, which coincides with the cleavage of Caspase 3. DNAPKcs autophosphorylation at Ser2056 results in inactivation of its kinase activity and DNA repair ability (44–46). These results suggest significant effects of [Flu+Clo+Bu+SAHA+Ola] on the complex interactions among proteins involved in the DNA damage response, DNA repair, cell cycle, and programmed cell death.

To further analyze the possible interaction between DNAPK and P53, MV4-11 cells were exposed to DNAPK inhibitors prior to addition of the chemotherapy drugs. The inhibitors Nu7741, LTURM34 and AZD7648 increased the level of P-DNAPKcs (S2056) but decreased the level of pan P53 relative to the control (i.e., no inhibitor) after the 24 h five-drug exposure (**Figure 6B**), suggesting that the stability of P53 was compromised in the presence of DNAPK inhibitors. These findings are consistent with DNA-PK phosphorylating and stabilizing P53 in this setting as previously reported (47, 48).

## 4 Discussion

We recently reported the *in vitro* synergistic cytotoxicity of Bu, gemcitabine (Gem), melphalan (Mel), SAHA and Ola in lymphoma cells (31). A clinical trial of [Gem+Bu+Mel+SAHA+Ola] as a pre-transplant (salvage) regimen for autologous HSCT for 35 patients with refractory Hodgkin’s and Non-Hodgkin’s lymphomas was very promising, yielding ~90% event-free and overall survival rates with a median follow-up of 16 months, without adding any discernible risk to patient safety by the addition of SAHA+Ola (32). The significant efficacy of such a combination of chemotherapeutic agents, HDACi, and PARPi in lymphoma prompted us to determine their activity in leukemia cells and the potential benefit of this combination as part of pre-transplant regimens in the context of patients with acute leukemia who typically receive allogeneic HSCT. Using the combination [Flu+Clo+Bu] as a backbone, which has proven to be an efficacious pre-transplant regimen for AML patients receiving allogeneic HSCT (2–5, 49), the HDACi SAHA and the PARPi Ola were added with the expectation of further enhancing drug cytotoxicity. Indeed, the present study shows a synergistic cytotoxicity of [Flu+Clo+Bu+SAHA+Ola] in AML cells, which may be attributed to a combination of drug-induced histone modifications, decreased protein PARylation, DNA damage, increased ROS production, decreased MMP, and down-regulation of the levels of DNA repair proteins.

**Figure 7:**
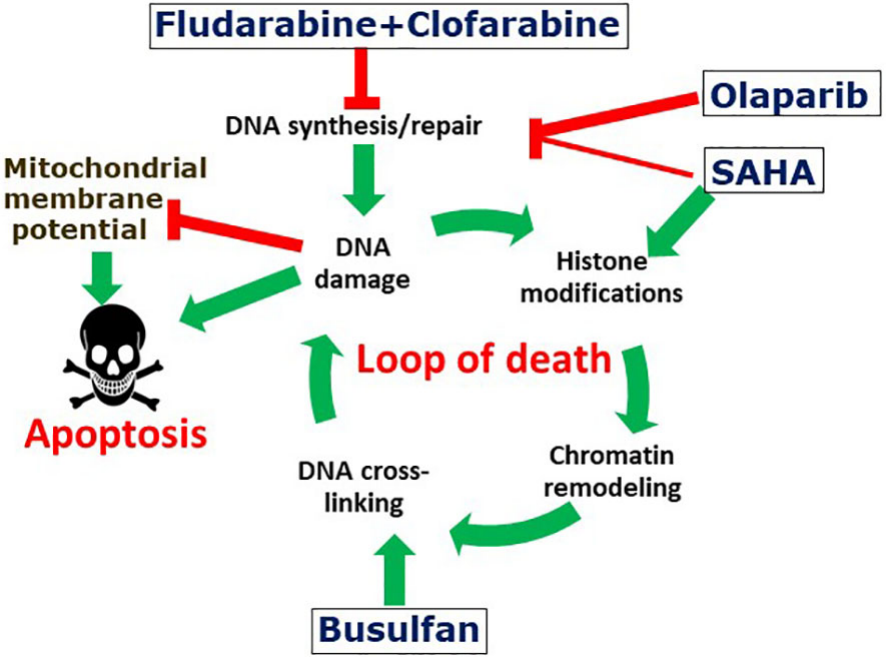
Suggested mechanism of the synergistic cytotoxicity of fludarabine, clofarabine, busulfan, SAHA and olaparib. The activated nucleoside analogs inhibit DNA synthesis and DNA repair, which results in complex DNA lesions. DNA strand breaks may cause histone modifications and chromatin remodeling, which may expose genomic DNA and make it potentially more accessible to DNA alkylation with busulfan. Attempts to repair DNA cross-links results in additional DNA strand breaks and the cycle is further amplified by additional DNA alkylations. The increased levels and complexity of DNA damage results in enhanced cell death. Olaparib and SAHA inhibit protein PARylation and histone deacetylation, respectively, resulting in inhibition of DNA repair and further enhancement of the cycle of DNA damage-repair and cell death. DNA damage is associated with decreased mitochondrial potential and consequently with increased apoptosis.

The low individual concentrations of Flu, Clo and SAHA used in these combination treatments with the expectation of ultimately minimizing patient toxicity in clinical trials may have provided the initial histone acetylation needed for chromatin remodeling, which in turn may have resulted in increased exposure of the DNA to Bu-mediated alkylation, as we described previously in the context of the so-called “Loop of Death” model for synergy between alkylating agents and nucleoside analogs in hematological malignancies (7, 50). The resulting DNA adducts may provide signals for additional histone acetylation to further open up the chromatin and increase the accessibility of the DNA to further alkylation, as per the “Loop of Death” model (**Figure 7**). These effects are presumably enhanced when Flu and Clo are incorporated into growing nascent DNA strands during replication via the same mechanism(s). This futile cycle of histone acetylation, DNA alkylation and nucleoside analog incorporation into DNA exacerbates DNA damage and potentially increases its complexity, making it more challenging for the DNA damage response to process correctly. This model does suggest the importance of drug sequencing for optimal anticancer efficacy, with the nucleoside analogs ideally being added prior to the alkylating agents. However, in the present study the cells were exposed concurrently to the various drugs, which thus represents a limitation in terms of its mechanistic implications.

Consistent with this model, the complex DNA damage invoked by [Flu+Clo+Bu] strongly activates the DNA damage response as indicated by increased phosphorylation of histone 2AX, a response that is even more strongly activated in combination with the HDACi and PARPi (**Figure 4**). Repair of the damaged DNA is known to require the initial binding of PARP1 at the damage site(s) (51), after which PARP1 PARylates itself, histones and certain chromatin-associated proteins to provide a scaffold for recruitment of the DNA repair machinery (52). This process could be abrogated by the combined effects of SAHA and Ola through inhibition of PARylation via PARP1 trapping to chromatin, a mechanism that explains the cytotoxicity of the HDACi trichostatin A and the PARPi talazoparib in leukemia cells (53). The DNA damage inflicted by [Flu+Clo+Bu] combined with the high levels of oxidative and replication stress characteristic of many human cancers may render these AML cells highly dependent on protein acetylation and PARylation, and therefore extremely sensitive to the added [HDACi+PARPi] exposure (**Figure 7**).

One disadvantage of using high dosages of chemotherapeutic agents is their known tissue toxicities. Our data show that combining low dosages of these drugs and agents with different mechanisms of action enhanced synergistic cytotoxicity against leukemia cells. Addition of SAHA and Ola to [Flu+Clo+Bu] significantly induced apoptosis as indicated by increased Annexin V positivity and activation of Caspase 3 (**Figures 2**, **3**). Since our clinical trial on [Gem+Bu+Mel+SAHA+Ola] in lymphoma resulted in minimal normal tissue toxicities, and notably with no cases of serious veno-occlusive disease (32), we anticipate that the [Flu+Clo+Bu+SAHA+Ola] combination will potentially result in low toxicities in AML patients undergoing HSCT. Further investigations, notably clinical trials, will be required to establish the therapeutic relevance of this drug combination. It should be noted however, that for such clinical trials, high-risk patients with relapsed/refractory AML and a low comorbidity index should be favored as the most optimal group in which to test the novel combination (4).

In summary, our data with [Flu+Clo+Bu+SAHA+Ola] provide evidence of interrelated mechanisms that commit AML cells to apoptosis following complex genotoxic insult. Since similar synergistic cytotoxicity of this 5-drug combination was observed in established AML cell lines and patient-derived cell samples, our results may be used as a hypothetical basis to justify a clinical trial to evaluate these drugs as part of pre-conditioning regimens for high-risk AML patients undergoing allogeneic HSCT.

## Data availability statement

The original contributions presented in the study are included in the article/supplementary material. Further inquiries can be directed to the corresponding author.

## Ethics statement

The studies involving humans were approved by Institutional Review Board of the UTMDACC. The studies were conducted in accordance with the local legislation and institutional requirements. The participants provided their written informed consent to participate in this study.

## Author contributions

BV: Conceptualization, Formal Analysis, Investigation, Methodology, Project administration, Supervision, Writing – original draft, Writing – review & editing. BY: Data curation, Investigation, Methodology, Writing – original draft. DM: Data curation, Writing – review & editing. JR: Conceptualization, Formal Analysis, Writing – review & editing. YN: Conceptualization, Formal Analysis, Writing – review & editing. UP: Conceptualization, Formal Analysis, Writing – review & editing. XT: Conceptualization, Formal Analysis, Writing – review & editing. BA: Conceptualization, Data curation, Formal Analysis, Funding acquisition, Investigation, Project administration, Resources, Writing – review & editing.
